# Rupture of Renal Transplant

**DOI:** 10.1155/2015/686584

**Published:** 2015-01-21

**Authors:** Shona Baker, Maria Popescu, Jacob A. Akoh

**Affiliations:** Department of Surgery, Derriford Hospital, Plymouth Hospitals NHS Trust, Plymouth PL6 8DH, UK

## Abstract

*Background*. Rupture of renal allograft is a rare and serious complication of transplantation that is usually attributed to acute rejection, acute tubular necrosis, or renal vein thrombosis. *Case Presentation*. LD, a 26-year-old male with established renal failure, underwent deceased donor transplantation using kidney from a 50-year-old donor with acute kidney injury (Cr 430 mmol/L). LD had a stormy posttransplant recovery and required exploration immediately for significant bleeding. On day three after transplant, he developed pain/graft swelling and another significant haemorrhage with cardiovascular compromise which did not respond to aggressive resuscitation. At reexploration, the renal allograft was found to have a longitudinal rupture and was removed. Histology showed features of type IIa Banff 97 acute vascular rejection, moderate arteriosclerosis, and acute tubular necrosis. *Conclusion*. Possible ways of avoiding allograft rupture include use of well-matched, good quality kidneys; reducing or managing risk factors that would predispose to delayed graft function; ensuring a technically satisfactory transplant procedure with short cold and warm ischemia times; and avoiding large donor-recipient age gradients.

## 1. Introduction

Rupture of renal allograft is a rare and serious complication of transplantation that is usually attributed to acute rejection, acute tubular necrosis, or renal vein thrombosis [1–3]. This usually occurs during the first 2-3 weeks following transplantation, but cases occurring as late as 65 months have been reported [4]. The clinical picture comprises hypotension, graft swelling, and pain, with a significant drop in haemoglobin (Hb). Treatment consists of immediate resuscitation and often an urgent nephrectomy; however, graft salvage by repair has been performed [5–9]. This report adds another case of early allograft loss due to irreparable rupture to the literature and highlights ways of avoiding the complication.

## 2. Case Presentation

LD, a 26-year-old male with chronic kidney disease stage 5 secondary to Alport's syndrome, was seen in the transplant clinic in February 2012 during the predialysis phase. He was deemed suitable for transplantation and was put on the waiting list once his eGFR fell below 15 mL/min. He suffered from hypertension requiring Amlodipine, Lisinopril, and Doxazosin and was also taking Omeprazole and Calcichew. LD had negative serology for Hepatitis B, Hepatitis C, CMV, HIV, and VDRL syphilis but positive for EBV and chickenpox. He had a 5-year pack history of smoking; however, his exercise tolerance was excellent. He started peritoneal dialysis in June 2012.

LD underwent renal transplantation in January 2014 with a kidney from donation after brain death (DBD). The donor was a 50-year-old female who died from hypoxic brain injury due to hanging and had a creatinine (Cr) of 430 mmol/L. The tissue mismatch was 110. Pretransplant biopsy of the sister kidney at another centre showed severe acute tubular injury. The transplanted kidney was initially slow to perfuse but this improved as soon as the systolic BP was raised to 150 mmHg. At perfusion, the cold ischaemia time was 14 hours and 20 minutes and the anastomotic time was 37 minutes.

In the immediate postoperative period, LD had a significant bleeding of approximately 1120 mL and he was therefore taken back to theatre for exploration. At surgery, bleeding was noticed from a small branch of the renal artery where a surgical clip had come off. The bleeding artery was ligated and haemostasis was achieved. Blood clots were evacuated and the wound washed out and closed. As a result of the haemorrhage, his Hb dropped from 86 to 68 g/L and he was transfused with four units of red blood cells and two units of fresh frozen plasma. Subsequently, LD developed hyperkalaemia which was treated successfully with insulin and dextrose. There was no significant urine output.

**Figure 1:**
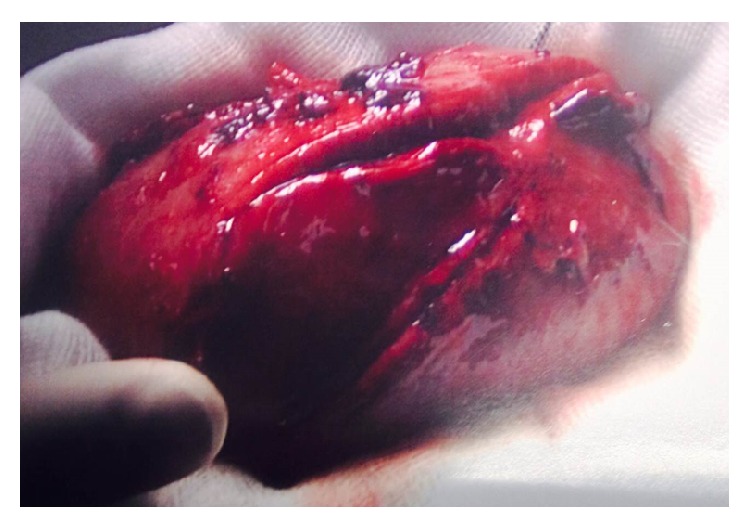
Explanted kidney showing a longitudinal rupture on the convex surface.

On the first postoperative day, his general condition was satisfactory though about 320 mL of blood was lost via his Robinson's drain. A Doppler-ultrasound scan showed a well perfused transplant kidney in all areas with good diastolic flow. Due to a high blood pressure of 214/136 mm/Hg, he was commenced on a higher dose of Doxazosin on the second postoperative day. His urine output improved but without evidence of transplant function and only 50 mL of blood drained via his Robinson's drain. On the third postoperative day, LD had a significant haemorrhage having lost 950 mL via his Robinson's drain and dropping his Hb from 78 to 53 g/L with cardiovascular compromise. He did not respond appropriately to fluid resuscitation and a further blood transfusion. He also developed pain and swelling over the transplant site. An urgent CT angiogram was performed which showed a large perinephric haematoma surrounding the transplant kidney but no active contrast extravasation. He was therefore reexplored. Intraoperatively, a longitudinal rupture of the kidney was identified (Figure 1) with bleeding from arterial anastomosis and the stem of the renal artery. The allograft was removed.

The explanted kidney was swollen and had an oblique rupture extending from the upper pole for 95 mm. Histological examination showed features of type IIa Banff 97 acute vascular rejection, moderate arteriosclerosis, and acute tubular necrosis. There were no features of thrombotic microangiopathy and C_4_d immunostaining of peritubular capillaries was negative. He recovered well and was discharged home on the 10th postoperative day.

## 3. Discussion

This case raises several important issues particularly in the context of the increasing use of marginal donor kidneys for transplantation as a way of addressing the global organ shortage. In a review of a large registry data, Ojo et al. [10] showed that transplantation using marginal donor kidneys was associated with a substantial reduction in mortality and improvement in life expectancy when compared to those remaining on dialysis. The transplanted kidney was from a donor with acute kidney injury (Cr 430 mmol/L) who was 24 years older than the recipient and in the absence of graft conditioning; a period of delayed graft function was expected. Given the potentially better haemodynamic condition of a younger recipient, it was felt that this donor kidney with features of acute injury would recover sufficiently to provide good function. The occurrence of this extremely rare complication is however not totally surprising. Older kidneys are known to have a negative outcome on renal transplantation as does a high donor-recipient age gradient [11]. It has been shown that a donor-recipient age gradient of more than 20 years is significantly correlated with worse 10-year graft survival [12]. Kidneys from donors older than 55 years of age showed significantly compromised graft outcomes when transplanted into recipients younger than 30 years of age [12].

LD suffered a stormy posttransplant progression with severe haemorrhage, resulting in hypoperfusion of the transplanted kidney adding to the ischaemic insults which no doubt caused further deterioration in the quality of the allograft. Though the mismatch between donor and recipient was 110 (which is considered to be a good match), the tacrolimus level on the third day after transplantation was suboptimal at 5.4 and it was not entirely surprising that features of acute rejection were reported on the explanted kidney.

As demonstrated in this case, ruptured renal transplants usually present as an acute event with graft pain, hypotension, and swelling in the graft area. Treatment involves initial supportive management and either nephrectomy or graft salvage. Although graft salvage is possible, preservation of renal allografts following spontaneous rupture is a surgical challenge and, despite successful repair, transplant nephrectomy might still be required [5, 13]. In our case, the decision to perform a nephrectomy was based on multiple factors: the use of an extended criteria kidney estimated to suffer from prolonged delayed function; the multiple postoperative complications which deteriorated further the quality of the graft; and the haemodynamic instability of the patient despite aggressive resuscitation. Susan et al. [14] reported successful salvage in all four cases of early allograft rupture due to acute rejection concluding that transplant nephrectomy can be avoided except in the presence of uncontrollable haemorrhage. Finley and Roberts [9] reported their experience of 22 renal allograft ruptures in 4418 transplants over a 20-year period. They were successful in salvaging 14 of the 22 ruptured transplants noting that a good human leucocyte antigen match and living kidney donor were reliable predictive factors for salvage, again emphasizing the importance of good quality of organs. However, the outcome of salvaging attempts is shown to be rather poor in the literature. Even with successful salvage operations, often the patient ultimately requires a nephrectomy [5, 13]. As described before, in those patients whose haemodynamic status cannot be stabilised by appropriate aggressive haemodynamic support therapy, graft nephrectomy should be considered the only definitive treatment [15].

Possible ways of avoiding allograft rupture include use of well-matched, good quality kidneys; reducing or managing risk factors that would predispose to delayed graft function; ensuring a technically satisfactory transplant procedure with short cold and warm ischemia times; and avoiding large donor-recipient age gradients.
